# “Alone Again, Naturally”: Mental Health Problems, Level of Personality Functioning, Social Withdrawal and Loneliness in Adolescents Admitted as Acute Inpatients in the Aftermath of the COVID-19 Pandemic

**DOI:** 10.3390/children10111743

**Published:** 2023-10-27

**Authors:** Johannes Boettcher, Dennis Radzuweit, Marie Mey, Philipp Rauch, Andreas Kogler, Claus Barkmann, Kirstin Goth, Sarah Hohmann, Carola Bindt, Ursula Voelker

**Affiliations:** 1 Department of Child and Adolescent Psychiatry, Psychosomatics and Psychotherapy, University Medical Center Hamburg-Eppendorf, 20246 Hamburg, Germany; 2 Department of Child and Adolescent Psychiatry, Psychiatric University Clinics, 4002 Basel, Switzerland; 3 Department of Child and Adolescent Psychiatry, University Clinics Saarland, 66421 Homburg, Germany

**Keywords:** adolescence, severe mental health problems, level of personality functioning, social withdrawal, loneliness

## Abstract

(1) Background: Adolescents admitted as acute inpatients belong to a particularly psychosocially vulnerable population. This study aimed to examine the clinical characteristics of an affected population in Germany using a theory-based approach. (2) Methods: We assessed the mental health problems, levels of personality functioning, and the severity of social withdrawal and loneliness in *n* = 62 adolescents admitted to an acute psychiatric inpatient unit. Cases were investigated cross-sectionally utilizing standardized psychometric questionnaires from the perspective of the patients and clinical experts. (3) Results: Mental health, level of impaired personality functioning, social withdrawal, and loneliness were all positively associated with the need for acute admission. Further analyses revealed that the level of personality functioning fully mediated the positive association between social withdrawal and mental health problems. In contrast, level of personality functioning only partially mediated the positive association between loneliness and mental health problems. (4) Conclusions: Our results suggest that more impairment in personality functioning might lead to poorer mental health when adolescents socially withdraw in the aftermath of the COVID-19 pandemic. Loneliness, social withdrawal, and the level of personality functioning may help identifying essential characteristics of adolescents admitted to acute psychiatric inpatient units and guide the development of specific interventions.

## 1. Introduction

Acute psychiatric inpatient units play an important role in adolescent psychiatry. Although it is well established that adolescents who seek acute psychiatric care often withdraw socially and feel lonely [1], little is known about the clinical characteristics and mental health problems of those admitted. This holds, especially in the aftermath of the COVID-19 pandemic, which posed a major challenge to adolescents [2] and vulnerable groups [3]. Moreover, recent studies support the assumption that the COVID-19 pandemic has impacted adolescents, including a short- and long-term deterioration in mental health, with increased depression, anxiety, psychological distress, and loneliness [4]. In Germany, acute psychiatric inpatient units for adolescents provide intensive treatment settings, optionally with locked doors. The main goal of these units is to stabilize individuals by reducing acute psychiatric symptoms and endangerment of self and others, often implying psychotherapeutic, psychosocial, and pharmacological interventions to overcome emotional crisis as well as symptoms such as social withdrawal and loneliness [5].

In the biopsychosocial model [6], mental health problems “are conceptualized as existing at a number of interacting, hierarchical levels from biological through to psychological and social levels” [7]. Social withdrawal is “an individual’s voluntary self-isolation from familiar and/or unfamiliar others through the consistent display of solitary behaviors” [8]. In contrast, loneliness is a “distressing feeling that accompanies the perception that one’s social needs are not being met by the quantity or especially the quality of one’s social relationships” [9]. Although social withdrawal and loneliness are inseparable, it is possible to feel lonely without being socially withdrawn, as is the reverse. A theoretical framework that explains perceived loneliness is offered by the *Evolutionary Theory of Loneliness* [10]. Humans are inherently social beings who desire to connect with others [11]. On the one hand, when the need for social connectedness cannot be satisfied, people may have an increased drive to remedy perceived relationship deficiencies and avoid the associated negative feelings [12]; on the other hand, people may be motivated to be vigilant and avoid potential social dangers or withdraw socially [10]. According to this theory, loneliness is a signal for self-preservation that leads to different behavioral and physical adaptations, which may include increased affective symptoms [10], possibly leading to severe emotional crises.

The *Crisis Theory* [13,14] can be used as a theoretical framework to understand the process and nature of emotional crises that lead to acute psychiatric episodes. According to the model, the crisis is preceded by a threatening situation, which can be developmental (e.g., the identity crisis of adolescence) or accidental (e.g., injury) [14]. In addition, vulnerability and protective factors are crucial for the individual’s perception of the threat and the ability to implement problem-solving behavior. If there is no timely improvement, a maladaptive state develops so that no helpful strategies can be applied, which in the model is called a breaking point [13]. As proposed by previous research, the model offers a way to better understand adolescents in a severe crisis within acute psychiatric settings [15]. In this context, the association between loneliness and mental health problems in adolescents has been theoretically linked to maladaptive personality characteristics [16]. The latter can be described by the level of personality functioning, which is the entry criterion for the alternative model for personality disorders [17,18]. According to the model, the level of personality functioning represents the core feature of all personality disorders, which are defined by impairments in self-related (identity and self-direction) and interpersonal (empathy and intimacy) functioning.

Recently, more quantitative research has been conducted on adolescents admitted to an acute psychiatric inpatient unit, focusing on the effectiveness of these units [19]. Studies examining adolescents in acute psychiatric inpatient units found symptom reduction in the constructs of mental health problems [20,21,22]. However, to our knowledge, quantitative research has not yet described this highly vulnerable group of adolescents at the level of symptoms of loneliness and social withdrawal. This is surprising because loneliness [23] and social withdrawal [24] were found as risk factors for adults with severe mental illness, which increase the likelihood of psychiatric admission. Although the association between loneliness and mental health problems and their link to social–behavioral deficits in adolescents have been empirically investigated [25,26], it still remains unclear what role personality characteristics might have in this context. 

**Figure 1:**
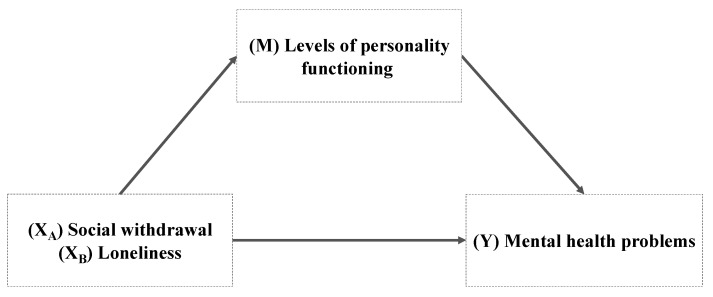
Predicted mediation models adapted from the from the Evolutionary Theory of Loneliness (Cacioppo and Cacioppo, 2018 [10]).

The objective of the current study was to investigate the clinical characteristics of adolescents admitted to an acute psychiatric inpatient unit in Germany in terms of exploring the following: (1) the severity of mental health problems and the agreement of patient and clinician ratings, (2) the severity of loneliness, social withdrawal, and levels of personality functioning, and (3) the association between the aforementioned variables. Additionally, we aimed to (4) explore the fit of a mediator model using a theoretical framework of loneliness and mental health problems possibly mediated by self-perceived personality characteristics in the form of levels of personality functioning (see Figure 1).

## 2. Materials and Methods

### 2.1. Study Design

In this study, a group of *n* = 62 adolescents was investigated cross-sectionally using standardized psychometric questionnaires between December 2021 and December 2022 at the Department of Child and Adolescent Psychiatry, Psychosomatics and Psychotherapy of the University Medical Center Hamburg–Eppendorf (UKE). The acute psychiatric inpatient unit treats patients up to the age of 18 years who are in acute psychiatric crises and require emergency admission. This specialized unit is locked and provides seven beds, offering a protective and supportive environment for inpatients who require emergency care. The acute psychiatric inpatient unit covers parts of Hamburg and in exceptions surrounding federal states in Germany. The study received ethical approval from the Medical Chamber Hamburg (2021-100618-BO-FF) and was preregistered in ClinicalTrials.gov (NCT05162287).

### 2.2. Variables and Measures

Mental health problems: The German version of the Health of the Nation Outcomes Scales for Children and Adolescents (HoNOSCA-D) [27,28] was used to assess the mental health problems and their severity of affected adolescents. The HoNOSCA-D consists of 13 items, which are answered on a five-point rating scale. The instrument comprises four scales of symptoms and functioning, including Behavior, Impairments, Symptoms, and Social. Additionally, a total score can be calculated by summing up the individual items, which range between 0 and 52, representing overall severity. Higher scores indicate a greater severity of problems. The HoNOSCA-D was administered by the patients [27] and clinicians [28]. The clinicians conducting the HoNOSCA had over three years of clinical experience and were trained according to the manual. Additionally, regular meetings were held to ensure a high level of consensus between raters. The HoNOSCA-D has shown good psychometric properties [27,28].

Social withdrawal: The Youth Self-Report (YSR; [29]) is a self-report measure of emotional and behavioral problems. The YSR comprises 112 items, which are answered on a three-point rating scale. We solely used five items of the subscales *Withdrawn/Depressed* to measure social withdrawal, as suggested by Barzeva et al. (2019) [8]. The same authors found good psychometric properties for using the YSR social withdrawal items [8].

Loneliness: The German version of the Three-Item Loneliness Scale [30] was used to assess self-reported loneliness in adolescents between 12 and 18 years. The instrument consists of three items, which are answered on a five-point rating scale. In the Three-Item Loneliness Scale, a total sum of all items can be formed to represent a general severity of loneliness. Higher scores indicate greater loneliness, with scores ranging from 0 to 12. Additionally, as suggested by Klein et al. (2021) [30], the total sum can be classified into the following three groups: 0 = not lonely, ≤3 = minor feeling of loneliness, ≥3 = lonely. The psychometric properties of the German Version of the Three-Item Loneliness Scale are considered to be good [30]. 

Level of Personality Functioning: The German version of the Levels of Personality Functioning-Questionnaire 12–18 (LoPF-Q 12–18) [31] was used to assess self-reported impairments in the levels of personality functioning in adolescents between 12 and 18 years. The level of personality functioning is part of the dimensional approach to personality disorders in the new ICD-11 and DSM-V classification system [32]. The instrument consists of 97 items, which are answered on a five-point rating scale. The LoPF-Q 12–18 contains four scales: Identity, Self-Direction, Empathy, and Intimacy. In this study, solely the total sum of all items was used to represent a general severity of impairment in personality functioning. Higher scores indicate greater impairment. The psychometric properties of the LoPF-Q 12–18 are considered to be good [31]. 

Socio-demographic and clinical variables: Clinicians completed a study-specific sociodemographic questionnaire about the patient’s sex, age, education level, family and living situation, psychiatric history in the family, and clinical variables. Clinical variables of the adolescents included the length of stay, voluntariness and reason for admission, readmission and associated number within the survey period, primary clinical diagnoses, psychological and somatic comorbidities, and the presence of non-suicidal self-injurious behavior or a history of attempted suicide. Furthermore, the present intake of psychotropic drugs and related classes were collected.

### 2.3. Sample

Patients aged 12 to 17 years who were admitted to the acute psychiatric inpatient unit and who fulfilled criteria of a psychiatric disorder (ICD-10-GM-2016: F10-F90) were included in the study. We excluded patients with organic, including symptomatic, mental disorders (F00-F09), severe physical and cognitive impairment (IQ < 70), lack of German language skills, uncorrected severe visual or hearing impairment, and adolescents with a length of stay under 24 h. The adolescents and their parents gave signed informed consent. The participants were allowed to withdraw from the study at any given time. 

**Figure 2:**
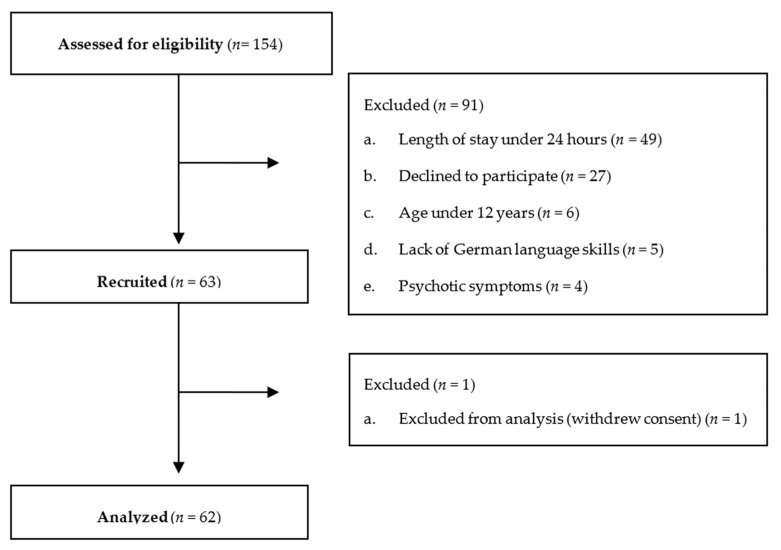
CONSORT flow diagram.

Between December 2021 and December 2022, one hundred and fifty-four adolescents were admitted. The data acquisition thus falls into the period after two lockdowns that took place in Germany due to the COVID-19 pandemic [33]. Questionnaires were handed out to 109 adolescents. A total number of 63 adolescents completed the questionnaires. The response rate was 57.8%. Finally, written consent from 62 adolescents was obtained. Figure 2 presents the CONSORT flow diagram.

### 2.4. Statistics

Description of the sample was performed using descriptive statistics (frequencies, means, and standard deviations) and bivariate tests (chi-square tests). Group differences were examined using Welch’s *t*-tests. An intraclass correlation (ICC) was conducted between self-ratings and clinician ratings. Associations between variables were analyzed with Pearson correlations. Cohen’s *d* was used as an indication of effect size. The level of personality functioning was used as the mediator of the relations between (A) social withdrawal and (B) loneliness and mental health problems. The mediations were tested following the procedure from Hayes [34]. The mediation analyses were conducted using the PROCESS macro, with 10,000 bootstrapping resamples, and bias-corrected 95% confidence intervals. $R^{2}$ was used as an indication of effect size. Statistical significance was set at *p* < 0.05 (two-tailed). Statistical analyses were conducted using SPSS Statistics 26.

## 3. Results

### 3.1. Characteristics of the Study Population

Table 1 presents the main sociodemographic and clinical characteristics. Regarding the adolescent’s age, there was no significant difference between participants and non-participants. Moreover, the gender between participants and non-participants did not differ significantly.

### 3.2. Mental Health Scores during Admission

Table 2 shows the mental health outcomes during admission for the self- and clinician-reported data. Patient self-ratings were significantly higher than clinician ratings except in the subscales Physical illness, disability, Peer relationship problems, and Family problems. Moreover, in the subscale Emotional symptoms, the patient self-rating was significantly lower than clinician ratings. Effect sizes ranged from trivial to large. Subsequent analyses indicated that the concordance of patient self-ratings and clinician ratings (ICC = 0.01–0.67) was poor to substantial.

### 3.3. Bivariate Associations

Table 3 shows a uni- and bivariate distribution of variables in the prediction model. No significant association was found for age with any other variable. Sex was significantly associated with loneliness and mental health problems with females being significantly lonelier and having more mental health problems. Social withdrawal, loneliness, level of personality functioning, and mental health problems were significantly associated with one another.

### 3.4. Mediation Analyses

**Figure 3:**
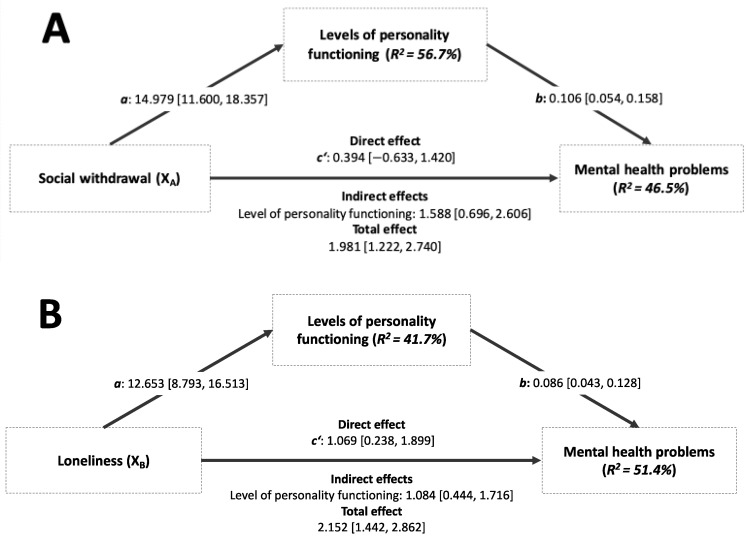
Mediation of social withdrawal (**A**) and loneliness (**B**) as predictors in adolescents admitted to an acute psychiatric inpatient unit.

Table 4 and Figure 3 show the mediation models for adolescents with social withdrawal and loneliness as predictors. The results revealed significant indirect effects of social withdrawal on mental health problems through the level of personality functioning in adolescents (Model A; see Figure 3). While the total effect of social withdrawal on mental health problems was significant (*b* = 1.981, *p* < 0.001, $R^{2}$ = 0.312), the direct effect was not statistically significant after including the level of personality functioning. The pattern of direct, indirect, and total effects suggests that the level of personality functioning fully mediates the association between social withdrawal and mental health problems in adolescents admitted to an acute psychiatric inpatient unit. The mediator model explained over forty-five percent of the variance in the population’s self-perceived mental health problems.

Regarding the model with loneliness as a predictor, results showed significant indirect effects of loneliness on mental health problems through the level of personality functioning in adolescents (Model B; see Figure 3). Meanwhile, the total effect of loneliness on mental health problems was significant (b = 2.152, *p* < 0.001, $R^{2}$ = 0.355); additionally, the direct effect was statistically significant after including the level of personality functioning. The pattern of direct, indirect, and total effects suggests that the level of personality functioning partially mediated the association between loneliness and mental health problems in adolescents admitted to an acute psychiatric inpatient unit. The mediator model explained over fifty percent of the variance in the population’s perceived mental health problems.

## 4. Discussion

The COVID-19 pandemic has been challenging for adolescents worldwide, especially for those vulnerable to mental health problems [3,4]. The current study used the theoretical framework of the *Evolutionary Theory of Loneliness* [10] to investigate the associations between social withdrawal/loneliness, levels of personality functioning, and mental health problems in adolescents who were seeking acute psychiatric help in the aftermath of the COVID-19 pandemic.

Adolescents admitted to our acute psychiatric inpatient unit mainly reported significantly different mental health problems compared to clinician ratings with poor to fair agreement in most domains. These findings are consistent with previous research on the instrument HoNOSCA [35,36,37]. The low concordance between patient and clinician ratings is especially found in inpatient samples, although concordance was better in outpatient adolescents [36,37]. Such results can be expected because instruments that rely on information from different classes of informants tend to have lower levels of agreement than those relying on informants from the same class [37]. Another explanation may be that clinicians underestimate adolescents’ mental health problems. Although the clinicians’ scores were similar to those in other studies [20,28,35], this can only be partially shown for the patient self-ratings, with ratings being relatively high [36,38]. These results on the part of the self-report may be particularly attributed to acute psychiatric inpatients and associated distress of the respective adolescents, who may arguably have the best insight into their own selves.

Most relevant, the findings of this study provide an understanding of the association between social withdrawal, loneliness, and mental health problems in adolescents, emphasizing the mediating role of personality characteristics represented by the level of personality functioning. Our mediation analysis supports this conclusion with social withdrawal but not loneliness. The level of personality functioning fully mediated the association between social withdrawal and mental health, whereas regarding loneliness, the results indicate a complementary mediation [39]. These findings support the hypothesis that more impairment in personality functioning leads to inappropriate adjustment when adolescents socially withdraw. Importantly, the results must be considered in light of the COVID-19 pandemic, during which social isolation among adolescents increased markedly and was associated with a significant reduction in well-being [40], the latter being linked to specific personality traits [41]. Consequently, the *Evolutionary Theory of Loneliness* in adolescents admitted to an acute psychiatric inpatient unit seems to be confirmed for social withdrawal and partially for loneliness. One reason why social withdrawal, and not loneliness, might have a better fit as a construct in the models is that the items on loneliness ask about emotions, which may be difficult to clearly experience, reflect on, and to differentiate for adolescents, who tend to have a more dysfunctional personality structure, whereas describing one’s social withdrawal as a more behavioral aspect might be easier to detect and describe [42]. Therefore, these findings are consistent with previous research investigating loneliness and mental health problems with other social–behavioral deficits [25,26].

Our results underline the relevance of factors to consider in adolescents admitted to acute psychiatric inpatient units. Although the framework within the *Evolutionary Theory of Loneliness* could not be confirmed for loneliness, preliminary analysis showed associations among the included variables. Future studies should investigate additional factors to identify more pertinent variables for loneliness within the framework. For example, identifying potential social cognitive factors such as age, gender, social self-efficacy, and unresolved attachment may be suitable additional variables in the theoretical model, as previous theoretical [16] and empirical research [25,26,43] suggests.

Although social withdrawal as well as feelings of social isolation undoubtedly play an important role in the mental health of adolescents admitted to acute psychiatric units, our results suggest that special attention should be paid to personality functioning levels in this context. Accordingly, beyond the interventions already known [44], future interventions to reduce loneliness and social withdrawal in adolescents should specifically distinguish between transient (state) and persistent (trait) loneliness, which can be well identified by the impairment of personality functioning. Moreover, in a preventive approach, adults close to adolescents such as teachers and healthcare professionals [45] should have the necessary resources and skills to identify social withdrawal and isolation and consequently provide low-threshold services early on to avoid the need for an admission to acute psychiatric inpatient units.

### Limitations

Due to the longer study duration and demands of patient data collection, this study is based on a small sample size, which may limit generalizability. These difficulties were related to the tedious initial training of the staff involved. Nevertheless, the study group with adolescents admitted to an acute psychiatric inpatient unit represents a sample challenging to recruit due to the low capacity of the ward as well as the criterion to include only patients who have been admitted for at least 24 h. Second, the sample may not be representative regarding the length of stay, the number of readmissions, and past suicide attempts of the affected adolescents. Since participants had a considerably longer length of stay, a higher number of readmissions, and more suicide attempts in the past compared to non-participants, non-response bias cannot be excluded. Consequently, the psychosocial burden may be overestimated, as adolescent participants were more impaired than non-participants. Nevertheless, the response rate was similar to prior research on child mental health conducted in Western countries before and during the COVID-19 pandemic [33]. Third, no causal conclusions can be drawn based on the cross-sectional mediation analyses. As a result of the one-group study design, it is also not possible to answer what effect the COVID-19 pandemic had on the results. Fourth, it is important to emphasize that the possible protective factor of previous or current outpatient psychotherapeutic treatment of adolescents could not be considered, which may influence the measured psychosocial variables. Finally, the questionnaires were distributed during the stay of the patients. Due to the number of questionnaires and the sometimes short stay of the patients, we cannot consider the exact time when the questionnaire was filled out between admission and discharge. Thus, there may be a temporal bias with significantly higher reports of mental health problems at the time of admission compared to discharge [20,38].

## 5. Conclusions

Our results contribute to the research on adolescents admitted to an acute psychiatric inpatient unit by describing mental health problems, loneliness, and the level of personality functioning of those admitted. Moreover, our results suggest that healthcare professionals may primarily focus on increased social withdrawal and impaired levels of personality functioning to stabilize adolescents admitted to acute psychiatric units. Thus, potential areas for therapeutic intervention can be identified to appropriately support affected individuals by counteracting social withdrawal via social–psychiatric and psychotherapeutic interventions during the stay and aftercare. Since the results were generated during and in the aftermath of the COVID-19 pandemic, a follow-up survey should be conducted to examine whether impairment in the surveyed constructs continues to be affected to this degree in such a vulnerable population. In addition, future research should examine specific short-term interventions in acute psychiatric settings focusing on social withdrawal and loneliness and personality functioning.

## Figures and Tables

**Table 1:** Sociodemographic and clinical characteristics of participants and non-participants.

Characteristics	Participants (*n* = 62)		Non-Participants (*n* = 92)		Test of Differences between Both Groups
	*M*	*SD*	*M*	*SD*	*p*
Patient’s age (years)	15.4	1.34	15.5	1.92	0.799
School grade	9.1	1.62	8.8	2.01	0.372
Length of stay (days)	24.2	52.20	5.2	8.07	0.006
Number of readmissions	1.6	3.5	0.6	0.83	0.030
	*n*	%	*n*	%	*p*
Gender					0.222
Male	16	25.8	25	27.2	
Female	44	71.0	67	72.8	
Diverse	2	3.2	0	0.0	
Education					0.768
Student	57	91.9	83	90.2	
Trainee	0	0.0	1	0.1	
Dropout	1	1.6	3	3.3	
Other	4	6.5	5	5.4	
Family situation					0.001
Parents together	28	45.2	34	37.0	
Parents divorced	17	27.4	2	2.2	
Parents separated	17	27.4	48	52.2	
At least one parent deceased	0	0.0	3	3.2	
Adopted	0	0.0	5	5.4	
Living arrangements					0.001
With one parent	26	41.9	32	34.8	
With both parents	30	48.4	26	28.2	
Other	6	9.7	34	37.0	
Psychiatric history in family					0.750
Yes	43	69.4	66	71.7	
No	19	30.6	26	28.3	
Admission					0.908
Compulsory	51	82.3	75	81.5	
Voluntary	11	17.7	17	18.5	
Nonsuicidal self-injury					0.114
Yes	49	79.0	62	67.4	
No	13	21.0	30	32.6	
Suicide attempt in past					0.030
Yes	31	50.0	30	32.6	
No	31	50.0	62	67.4	
Psychiatric disorder (ICD-10)					
F10–F19	8	12.9	9	9.8	0.544
F20–F29	3	4.8	3	3.2	0.620
F30–F39	48	77.4	50	54.3	0.004
F40–F49	10	16.1	10	10.9	0.341
F50–F59	12	19.4	3	3.2	0.001
F60–F69	8	12.9	10	10.9	0.700
F70–F89	1	1.6	1	1.1	0.777
F90–F98	33	53.2	57	62.0	0.362
Number of psychiatric disorders					0.009
One	15	24.2	45	48.9	
Two	36	58.1	41	44.6	
Three	8	12.9	5	5.4	
Four	3	4.8	1	1.1	
Somatic disorder (ICD-10)					0.316
Existent	6	9.7	5	5.4	
Non-existent	56	90.3	87	94.6	
Medication at admission					0.223
Antidepressants	6	9.7	12	13.0	
Antipsychotics	12	19.4	21	22.8	
Anxiolytics, Sedatives, Hypnotics	0	0.0	2	2.2	
Phase prophylactics	2	3.2	0	0.0	
Other	1	1.6	3	3.3	
Multiple	17	27.4	14	15.2	
None	24	38.7	40	43.5	

Note. *M =* Mean, *SD =* Standard deviation. Comparison between groups was assessed using Welch’s *t*-tests or $\chi^{2}$ two sample tests.

**Table 2:** Distribution of the mental health problems for the self- and clinician-reported data of the HoNOSCA-D.

HoNOSCA Scales	Self-Ratings (SR)		Clinician Ratings (CR)		Test of Differences (Self vs. Clinician)	% of SR Score ≥ 3	% of CR Score ≥ 3
Disruptive, aggressive problems	1.9	1.48	0.9	1.01	0.80 (0.001)	40.3	8.1
Overactive, attention difficulty	2.9	1.20	1.4	0.98	1.40 (0.001)	72.6	11.3
Self-injury	3.1	1.30	2.3	1.27	0.63 (0.001)	75.8	53.2
Alcohol, drug misuse	1.0	1.41	0.5	0.94	0.56 (0.002)	17.7	4.8
Scholastic/language skills problem	3.0	1.29	1.3	1.10	1.42 (0.001)	67.7	11.3
Physical illness, disability	0.8	1.33	0.5	0.94	0.24 (0.123)	14.5	4.8
Hallucinations, delusions	1.7	1.56	0.6	1.09	0.80 (0.001)	37.1	8.1
Psychosomatic problems	1.9	1.48	1.2	0.94	0.59 (0.001)	37.1	6.5
Emotional symptoms	2.6	1.40	2.9	0.72	−0.31 (0.045)	59.7	85.5
Peer relationship problems	2.1	1.53	2.2	0.99	−0.04 (0.826)	43.5	40.3
Self-care, independence problems	2.7	1.32	0.9	1.04	1.50 (0.001)	64.5	8.1
Family problems	2.5	1.53	2.4	0.71	0.04 (0.806)	56.5	41.9
Poor school attendance	2.0	1.38	1.4	1.99	0.36 (0.041)	35.5	21.7
Externalizing problems	10.4	3.85	4.4	2.86	1.72 (0.001)	-	-
Emotional symptoms	17.6	7.58	11.0	3.71	1.08 (0.001)	-	-
Total score	28.0	10.45	18.3	5.79	1.14 (0.001)	-	-

Note. *M =* Mean, *SD =* Standard deviation. Comparison between groups was assessed with Welch’s *t*-tests. *d* = Cohen’s *d.* SR = Self-ratings, CR = Clinician ratings.

**Table 3:** Associations between predictor and outcome parameters (*n* = 62).

Variables	1	2	3	4	5	6
1. Age	-					
2. Sex	0.21 (0.100)	-				
3. Social withdrawal	0.02 (0.890)	0.10 (0.443)	-			
4. Loneliness	0.15 (0.431)	**0.43 (<0.001)**	**0.48 (<0.001)**	-		
5. Level of Personality Functioning	0.04 (0.745)	0.20 (0.113)	**0.75 (<0.001)**	**0.65 (<0.001)**	-	
6. Mental health problems	0.05 (0.685)	**0.33 (<0.009)**	**0.56 (<0.001)**	**0.62 (<0.001)**	**0.67 (<0.001)**	-
*M*	15.4	0.7	8.4	7.6	218.1	27.98
*SD*	1.34	0.45	2.95	2.99	58.63	10.45
*Median*	15.2	1.0	9	8	225.5	28.5
*Min, Max*	13, 18	0, 1	1, 13	0, 12	46, 314	5, 49

Note. Displayed values are Pearson *r*, with *p* values in parenthesis. Statistical significance was set at *p* < 0.05 and indicated in bold. Biological sex was used for this analysis to generate a binary variable. Sex: 0 = male and 1 = female. Social withdrawal was assessed with Youth Self-Report—Social withdrawal scale, Loneliness was assessed with the Three-Item Loneliness Scale, and Level of Personality Functioning was assessed with the Level of Personality Functioning Questionnaire 12–18 Total Scale. Mental health problems were assessed with the Health of the Nation Outcomes Scales for Children and Adolescents—Total scale.

**Table 4:** Mediator models predicting mental health problems in adolescents (*n* = 62).

Mediation Model A	LoPF-Q 12–18 (*M*)				HoNOSCA (*Y*)			
		*b*	*SE*	*p*		*b*	*SE*	*p*
YSR—Social withdrawal (X*_A_*)	*a*	14.979	2.331	<0.001	*c’*	0.394	0.755	0.604
LoPF-Q 12–18 (*M*)		-	-	*-*	*b*	0.106	0.026	0.003
Constant	*i_M_*	92.995	21.654	<0.001	$i_{Y}$	1.576	3.974	0.693
	*F*(1, 60) = 78.661, *p* < 0.001, *R*^2^ = 0.567				*F*(2, 59) = 25.654, *p* < 0.001, *R*^2^ = 0.465			
**Mediation Model B**	**LoPF-Q 12–18 Total (*M*)**				**HoNOSCA Total (*Y*)**			
		***b***	***SE***	***p***		***b***	***SE***	***p***
3-Item Loneliness Scale (X_B_)	*a*	12.653	2.271	<0.001	*c’*	1.069	0.409	0.011
LoPF-Q 12–18 Total (*M*)		-	-	*-*	*b*	0.086	0.025	0.001
Constant	*i_M_*	121.653	20.245	<0.001	$i_{Y}$	1.168	4.428	0.784
	*F*(1, 60) = 42.994, *p* = < 0.001, *R*^2^ = 0.417				*F*(2, 59) = 31.245, *p* < 0.001, *R*^2^ = 0.514			

Note. *b* = unstandardized regression coefficient, *SE* = standard error, 95%-CI = 95% confidence interval. YSR—Social withdrawal = Youth Self-Report—Social withdrawal scale, LoPF-Q 12–18 = Level of Personality Functioning Questionnaire 12–18—Total scale, HoNOSCA = Health of the Nation Outcomes Scales for Children and Adolescents—Total scale.

## Data Availability

The datasets generated during the current study are available from the corresponding author upon reasonable request.
